# The Homology of the Dental Tissues, Etc.

**Published:** 1877-06

**Authors:** A. H. Thompson

**Affiliations:** Topeka, Kansas


### SELECTIONS.

### THE HOMOLOGY OF THE DENTAL TISSUES, THEIR SUSCEPTIBILITY TO SUPPRESSIVE ECONOMY, AND THEIR PRESENT DEGENERACY IN MAN.

BY A. H. THOMPSON, D.D.S., TOPEKA, KANSAS.

### *Susceptibility to Suppressive Economy.*

THE homology of the dental tissues with the tissues of the derm, and the special identity of the enamel with the extra-vascular appendages, render it certain that they are governed by the same laws, are subject to the same influences, and possess the same phenomena of character as the allied tissues. Community of origin and similarity of structure and nature necessarily establish identity of the manner of life, of the methods of maintaining life with its varied phenomena, of similarity of service rendered to the economy and of the process of final dissolution and expulsion from the system. The relationship and homology of the teeth with the derm and its varied appendicular productions are established by demonstration. These appendages are as diverse in form and peculiarity of nature as tissues can well be, and yet preserve the unity of character which they do not fail to do. Teeth, spines, scales, dermal plates, feathers, nails, hair, bristles, horn, hoof, etc., varied in form and apparent purpose as tissues can well attain, are yet closely related in structure and function, with variations, of course, within certain limits.

The resemblances of character between the teeth and other dermal productions, to which our purpose at present directs our attention, are (1) the use and function of the teeth, and (2) their transient and inconstant nature. In these we claim that they are but related to and resemble the other structures of the derm.

(1) The use and purpose of all extravascular appendages is, primarily, the protection of the derm and the sub-dermal softer tissues against the friction, contact, or other deleterious influence of foreign substances. This is the self-evident purpose of the erection of all super-dermal structures. Extra dovelopment of these parts for special effectiveness in particular offices is secondary to the primary function of mere protection of the derm.

Epithelium is a universal and inseparable characteristic of the external surface of all skin, from the lining of a capillary to the sole of the foot, and is such by virtue of its power to resist the contact of substances foreign to the skin or the soft tissues beneath. Epithelium being present, and the demand for a tissue more dense arising to resist a certain external influence that promises to be deleterious to the skin and soft parts, the result, if life is to be maintained, would be the development from the epithelium, as the most external tissue, of an organ to meet the emergency. We can see how hair thus became developed as a protection against cold as a primary purpose. The epithelium was insufficient to protect the soft parts from the weather, and a new product, modified from the epithelium, arose. We can see how this product was further evolved for the secondary purpose, in many instances, of ornamentation by the influence of sexual selection, or how the color of the hair has been controlled by natural selection and developed into mimicry for protection against enemies or the capture of prey. Or again, as tactile organs to supplement the sense of touch. In birds we can see how a different dermal production, feathers, (possibly on account of reptilian affinities, feathers being more nearly related to reptilian dermal plates than to mammalian hair), were primarily evolved for the protection of the derm from the contact of water, and became subsequently modified for the purpose of aerial locomotion. Feathers are also subject to the secondary purposes of ornamentation and protective mimicry

(Wallace, "Contributions to Natural Selection"). In the case of the nails of the digits, which were apparently developed for the primary purpose of the protection of the derm in digging in the ground, we see greater effectiveness reached by special development for the purposes of seizing prey and climbing. Or in hoofs, protection is afforded the derm and speed facilitated by special modification and development of the nails, as a secondary purpose. In spines and dermal plates, we have, perhaps, the simplest form of protective organ, and which takes on somewhat of ossific or endo-skeleton structure and affinity. In some forms, however, as the scales of fish, the secondary purpose of ornamentation, etc, are attained, but as a rule these productions aspire to simple protection of the derm. In this they illustrate the primary object of the development of all dermal appendages as protective organs in their simplest purpose.

We therefore claim that the teeth have had the same purpose of organization, and that they were produced originally as mere dermal structures for the protection of the skin of the jaw against the friction of food in mastication, and were subsequently developed into organs of various forms in different species. This variety was developed for the purpose of special effectiveness in the peculiar use of each species in the work of reducing its particular food. This, we claim, is the story of their life. We see this illustrated by the resemblance between the teeth and spines of the skin as mere dermal protectors, as pointed out by Mr. C. S. Tomes (op. cit.), as follows: "Just as in the external skin bony or dentinal plates are developed for the purpose of protecting it from destruction by attrition, so for a similar purpose teeth are developed in that portion of the mucous membrane which covers the jaws."

The ossific form of dermal plate was developed very early in the history of animal life, and we find the teeth taking this form in the lowest organizations in being composed largely of bone or dentine. Both these tissues are found in dermal structures, and indicate their homology by the similarity of the circumstances of their occurrence.

Evolution of organic form, differentiation of systemic organization and of issue, the evolution of food, and the influence of food-selection (as a power to be regarded), led to the development of progressively higher forms of dental organization. But while the teeth have reached a very elevated specialization in many forms, they universally retain their primary office of the reduction of food as their especial function. This office of the teeth can only be regarded as a single manifestation of the function of all dermal structures,—the protection of the derm. From an elementary form which afforded the rudest protection to the gum-tissue of the jaw, all progressively higher and subsequent forms were evolved and elaborated. The first step taken, natural selection seized the modification and produced the varied forms of dentures we observe to-day. We can see how the now special function of forcible mastication arose and became elaborated, and how it was developed from a simple to a complex function.

Like other dermal structures, the teeth also perform other services to the economy and fill offices that are but secondary, but that are often very peculiar and conspicuous, and, indeed, often to the extent of overshadowing the prime function of mastication. This extreme is noticed in the tusks of the narwhal, elephant, etc., and other peculiar forms. Most frequently, however, the teeth still retain some effectiveness as prehensory and masticating organs. Among secondary services we may merely note their use as secondary sexual organs, and other battle purposes, and as auxiliaries in locomotion, vocalization, ornamentation, etc. In man the employment of the teeth for their prime purpose, mastication, is still apparent and present, but the function is disappearing, and prehension is, perhaps, totally aborted. As secondary organs they retain no function, perhaps, except those of vocalization and ornamentation; but these are, apparently, of the most conspicuous importance to the species, and are, possibly, not even second in value to mastication. Yet these functions being secondary, and not upheld by natural selection, having no natural force of sufficient power to maintain them for these services, the teeth will most probably disappear before the destructive effects of the rapid disuse of mastication. Desirable as it may be that the human teeth should be retained for the æsthetic purposes of vocalization and ornamentation, which the higher physical and psychical life of the species imposes upon them, artificial selection is powerless to resist the ultimate catastrophe of their total loss. Sexual selection is probably the only force to which we can look for their support as ornamental organs, for their loss would greatly mar the beauty of the female of the species, upon which propagation largely depends. Evolution will, of course, modify sexual taste by the time we reach the edentulous age, however. Yet when we observe what a wonderful development and elaboration of beauty has been wrought in the female of the civilized races of man by the force of sexual selection under civilization, as compared with savage races, we feel that this force ought to be sufficient to maintain the teeth in a form of beauty for the purposes of sexual ornamentation. But we can scarcely hope that the iconoclastic tide of the disuse of mastication may be checked or impeded. The intellectual use of the teeth for vocalization, desirable as their continuance for this purpose would be, is a force of infinitesimal weight. It therefore seems that the abortion of the primary function of the teeth, mastication, will necessarily induce their suppression, as being a force that cannot be resisted by the influence of the importance of secondary function.

(2) The next important resemblance of character between the teeth and other dermal productions which it is our purpose to notice, is their transient and inconstant nature—*i. e.*, that the teeth are, by virtue of their dermal relationship, in harmony with other dermic structures, subject to the phenomena of shedding and reproduction. This is exhibited in varying degrees of manifestation in different animals, but prevails to some extent in all. In the case of the dogfish, as cited by Mr. Tomes (op. cit.), the teeth and spines are continuous, and are shed and reproduced without cessation. He says that the spines "are much less often shed and reproduced" than the teeth, "so that it is less easy to find them in all stages of their growth. I believe, however, that they follow a course essentially similar to that of the teeth." Reptiles posses a constant succession of teeth. Most of the mammalia are diphyodonts (or "heterodonts"—Tomes),—*i.e.*, possess "a milk set of teeth which is displaced by a permanent set." Some are monophyodonts (or "homodonts"), (as edentata, cetacea, etc.), and produce but one set of teeth.

The fact to be brought out is that the teeth are shed and reproduced, no matter with how much dissimilarity in frequency, in all animals possessing teeth, in a manner identical with or similar to the production, casting off, and reproduction of other epithelial and dermal structures. From the evidence afforded us we feel warranted in advancing the hypothesis, that as the teeth partake of the transitory and inconstant nature of all dermal appendages, so are they most readily susceptible to the forces that induce the suppression of special parts when permitted by disuse and coerced by the law of economy of growth. They are, by virtue of their very nature, markedly and readily amenable to an extreme degree to the physiological forces which induce the disorganization in various ways of different parts of the economy. The teeth are thus acted upon, after being formed, by the absorption of the root, and of their supporting tissue, the alveolus, which induces their expulsion. In the formative processes they are subject to marked effects of various influences, both physiological and pathological, which produce phenomena of aberrations from the normal type of conspicuous importance. These include the various abnormal effects upon the teeth, and particularly upon the enamel, of exanthematous diseases (again indicating their dermal relationship), the effects of inherited diseases (which are mainly circulatory) ; the accidental and inherited suppression of certain teeth and extra-production of others,—which may be called infra-and supra-supplementary teeth, etc. These, among other manifestations of the capabilities of the teeth to direct suppression, either partial or total, evidence our meaning.

The teeth, like other dermal appendages, do not belong to the class of organs that are vital in their direct importance to the economy, and especially is this manifested in man. They can be altered in form and number in most species, readily and safely, and the species continue to exercise the ordinary functions of life. Profs. Huxley and Flower (*Nature*) have shown that the teeth are infinitely variable in form and susceptible to moulding influences, though persistent in tissual structure. Every species exhibits the effects of this susceptibility in which teeth have been lost or altered through changed habits concerning its food. In man the capacity for change has been brought to the extreme of his being able to lose any or every factor of the denture, and yet maintain life with ease by means of the artificial methods of reducing food which he possesses. This fact indicates his independence of teeth even in the present early stage of the progress of their suppression, and suggests his ultimate edentulous condition.

Previous articles of this series of papers have been devoted to the elucidation cf the fact that mastication is passing into disuse, and that the teeth are, in consequence, subject to and in course of suppression, like all useless organs, in obedience to irrevocable and irresistible law. We now endeaver to show that the teeth are, as tissues, most readily amenable to this economic suppression, and that this has been manifested in many striking cases, illustrating this impressibility by forces, normal and abnormal, that induce variations from the standard of normality in man. The most conspicuous and valuable of these cases of variation and which demonstrate our hypothesis of the homology of the teeth, are those which are accompanied by abnormal conditions of the hair, or nails, or both. Cases of collateral abnormality of these appendages in the same individual evidence this relationship and similarity of character. This has been noticed by Mr. Tomes (op. cit.), as before quoted in this paper (Part I,), and in the following instances which he mentions: "The famous hairy family of Burmah . . with silky hair developed over the face, transmitted this peculiarity to the third generation, and in each case the teeth were very deficient in number. The case of the hairy man and his son . . . from the interior of Russia . . . were also almost toothless. . . The hairy woman, Julia Plastrana, had unusually large teeth, . . and also hypertrophy of the gums and alveolar processes. . . . In the large teeth, the enormons papillæ of the gum, and the redundant hairs on the face, we have evidence of a disposition to hypertrophies of the integument, affecting in different places the different tegumentary appendages which happen to be there. And that the teeth are dermal appendages has been shown at a previous page." In the *British Journal of Dental Science* (1875) a case is noticed of deficiency of most of the teeth, which is accompanied by total absence of the toe-nails and rudimentary finger-nails. The following case came under the observation of the writer: Deficiency of the teeth was accompanied by a sparseness of beard which amounted to its almost total absence, although the hair and nails seemed natural. The patient (æt. about 28) did not remember losing any of the deciduous teeth except the first molars, and the denture presented the following condition: Inferior denture—bicuspids present, though separated by a space from the canine, small and flattish apex; incisors small and like deciduous teeth; canine small, round, and conical; first molars present, small; no other molars. Superior denture: incisors small and compressed; canines absent; first bicuspids small; one second molar present; other molars absent. This case of deficiency of hirsute covering, accompanied by deficiency in number and organization of the teeth, shows an interesting and valuable intersusceptibility of these related tissues. It does not matter that such phenomena are sometimes directly due to syphilis; the evidence is just the same. Mr. Geo. Barraclough (in the *Medical Times and Gazette*) speaks of the value of the susceptibility of the teeth to this disease in its diagnosis, and says, "I attach so much importance to the affections of the teeth in enabling us to ascertain the existence of hereditary taint, because of all syphilitic lesions they are the most constant. . . . That this constancy should exist and the affection be so well marked need not excite surprise when we consider that the teeth stand, as it were, structurally, half-way between the endo-and exo-skeletons." Farther on he speaks of the disease as affecting the hair and causing its loss, in a peculiar manner, exhibiting the sympathy maintained by the extra-vascular tissues. Thus the susceptibility of the teeth to abortive influences again and again points out their readiness to be affected by forces that cause the total or partial atrophy or loss of the dermal appendages, and the ease with which they are suppressed or altered in form.

Mr. Charles Darwin ("Variations of Plants and Animals under Domestication," 2d ed., 1876), speaking of the correlation of deficiency of the teeth and abnormal conditions of the hair, says, "With man several striking cases have been recorded of inherited baldness with inherited deficiency, either complete or partial, of the teeth. In an analogous case . . . that of a Hindoo family in Scinde, in which ten men, in the course of four generations, were furnished in both jaws taken together with only four small and weak incisor teeth, and with eight posterior molars; they have very little hair on the body, and become bald early in life. . . There is a . . . connection between the hair and teeth in those rare cases in which the hair has been renewed in old age, and has been usually accompanied by a renewal of the teeth." He gives instances, some of which we have noted, of redundancy of hair being accompanied by deficiency or redundancy of the teeth, all showing the close relationship of these tegumentary structures. Again, "There is a manifest relationship throughout the body between the skin and various dermal appendages, such as hair, feathers, hoofs, horn, and teeth. . . . Hairless dogs are deficient in their teeth . . . and men with redundant hair have abnormal teeth either by deficiency or excess."

In most of the cases of deficiency of the teeth, they present the condition of "arrested development." The teeth are frequently the deciduous ones remaining permanently, or the permanent erupting unpreceded by the deciduous set; but in each event they present, usually, the phenomena of imperfect and deficient organization. And what is remarkable and significant is, that the variations in the teeth of man from the normal type tend in the direction of *decrease* of perfection of organization. We do not see variation in the direction of greater perfection and efficacy in man, and rarely find dentures that we can consider as up to the standard of the normal type: but our experience in observation is quite the contrary. On this point Mr. Darwin (op. cit.) says (and quotes Mr. Romanes), "All parts are somewhat variable, and fluctuate round an average point. Now, when a part has already begun from any cause to decrease, it is very improbable that the variation should be great in the direction of increase, as of dimensions, etc. . . . The long-continued crossing of many individuals furnished with an organ which fluctuates in greater degree towards decrease than towards increase, will slowly but steadily lead to its diminution." Or, on final reduction, he says, "When a part becomes diminished by disuse prolonged through many generations, the principle of economy of growth, together with inter-crossing, will tend to reduce it still further." Deficiencies of teeth may be and are transmitted through the principle of inherited mutilations so often of marked character. In many families a single tooth, or pair of teeth, are hereditarily absent, which may have originated in a mutilation followed by accidental transmission in the first instance. In the singular cases of extensive deficiency before noted, one of the causes of transmission may have been the operation of this law.

But in all these and similar cases of partial or total suppression of the teeth, we see the peculiar impressibility which they, as extra-vascular appendages present, in common with other dermal productions. Without multiplying cases, for a few instances will illustrate a given meaning as well as a multitude, we submit that the teeth are, by nature of their homological relationship, amenable to an extreme degree to the laws that induce the suppression of a part when variation is desirable. This variation in the case of the teeth in man being in the direction of decrease of organization, the tendency is towards the result of final and inevitable abortion.—*Dental Cosmos.*

